# Magma mixing induced by particle settling

**DOI:** 10.1007/s00410-016-1305-1

**Published:** 2016-10-15

**Authors:** Christian J. Renggli, Sebastian Wiesmaier, Cristina P. De Campos, Kai-Uwe Hess, Donald B. Dingwell

**Affiliations:** 1 Department of Earth and Environmental Sciences, Ludwig-Maximilians-Universität München, Munich, Germany; 2 Research School of Earth Sciences, Australian National University, Canberra, Australia; 3 Department of Physics (Geology), GEOVOL, University of Las Palmas, Gran Canaria, Spain

**Keywords:** Magma mixing, Particle settling, X-ray microCT, Rhyolite–basalt, Liquid rope coiling

## Abstract

A time series of experiments at high temperature have been performed to investigate the influence of particle settling on magma mixing. A natural rhyolite glass was held above a natural basalt glass in a platinum crucible. After melting of the glasses at superliquidus temperatures, a platinum sphere was placed on the upper surface of the rhyolitic melt and sank into the experimental column (rhyolitic melt above basaltic melt). Upon falling through the rhyolitic–basaltic melt interface, the Pt sphere entrained a filament of rhyolitic melt in its further fall. The quenched products of the experiments were imaged using X-ray microCT methods. The images of our time series of experiments document the formation of a rhyolite filament as it is entrained into the underlying basalt by the falling platinum sphere. When the Pt particle reached the bottom of the crucible, the entrained rhyolitic filament started to ascend buoyantly up to the initial rhyolitic–basaltic interface. This generated a significant thickness increase of a comingled “melange” layer at the interface due to “liquid rope coiling” and piling up of the filament. As a consequence, the basalt/rhyolite interface was greatly enlarged and diffusive hybridisation greatly accelerated. Further, bubbles, originating at the interface, are observed to have risen into the overlying rhyolite dragging basalt filaments with them. Upon crossing the basalt/rhyolite interface, the bubbles have non-spherical shapes as they adapt to the differing surface tensions of basaltic and rhyolitic melts. Major element profiles, measured across the rhyolite filaments, exhibit asymmetrical shapes from the rhyolite into the basalt. Na and Ti reveal uphill diffusion from the rhyolite towards the interface in the filament cross sections. These results reveal the potential qualitative complexity of the mingling process between rhyolitic and basaltic magmas in the presence of sinking crystals. They imply that crystal-rich magma mingling may be expected to be accelerated with respect to crystal-poor systems. We urge the further fluid dynamic analysis of these phenomena, obtainable for the first time using detailed tomographic imaging.

**Electronic supplementary material:**

The online version of this article (doi:10.1007/s00410-016-1305-1) contains supplementary material, which is available to authorized users.

## Introduction

Magma mixing is the combined process of physical mingling and diffusive chemical mixing between end-member magmas. The mingling component of mixing is a physical dispersion of the magmas, whereas the diffusion is in response to the potential gradients of the diffusing species and acts to homogenise the mixture by decreasing chemical gradients (Perugini and Poli 2012). The combination of mingling (which enlarges melt interfaces) and chemical mixing (diffusion between melts) is summarised under the term chaotic mixing (Perugini and Poli 2000; Perugini et al. 2003; De Campos et al. 2011). Such mingling results in a chaotic, three dimensional dispersion of two coexisting fluids, forming active regions of the development of filaments and coherent regions reminiscent of enclaves (Perugini et al. 2002). The interfacial area increases exponentially by multiple stretching and folding of the liquids (Welander 1955). As a consequence of the chaotic nature of mingling, the structures are self-similar over several orders of magnitude (Perugini and Poli 2000). The degree of chaos depends on the mingling/mixing mechanism and rheological properties and forms variable structures of varying complexity.

Mingling is controlled by a range of processes. A main contributor is the density contrast yielding buoyancy forces between the end-member magmas due to differences in temperature, composition and volatile content (Turner and Campbell 1986; Oldenburg et al. 1989; Jellinek and Kerr 1999; Longo et al. 2006). As volatiles exsolve and rise buoyantly, bubbles can drag a denser magma into an overlying, more buoyant magma (Ruprecht et al. 2008; Wiesmaier et al. 2015). Additionally, the motion of crystals and xenoliths relative to the interface between two compositionally different magmas can drag one magma into another. It is this process which we have investigated experimentally here.

In recent years, chaotic magma mixing has been studied experimentally with natural silicate melts at high temperatures (Morgavi et al. 2013a, b, c). Those experiments studied the fate of the chemical components and explored their dependence on the chaotic mingling dynamics, yielding the conclusion that magmas with viscosity contrasts of up to four orders of magnitude can mix efficiently (Morgavi et al. 2013a, b, c). This observation stands in sharp contrast to earlier analyses which had led to the suggestion that the rheological differences between mafic and silicic magmas prohibit mixing (McBirney 1980).

The controlled addition of crystals or bubbles in these experiments has been a major challenge. Both bubbles and crystals have a major effect on the rheological behaviour of magmas. Depending on the crystal and bubble fractions in the magma, the viscosity increases considerably and makes rheological experiments at high temperatures difficult (Lejeune and Richet 1995; Costa 2005). Further, magma mixing experiments are commonly performed above the liquidus temperature of the magmas in order to allow reasonable experimental time scales. The behaviour of bubbles in experimental magma mixing has only recently been addressed (Wiesmaier et al. 2015). There, bubbles were observed to have moved from basaltic melt into an overlying rhyolitic melt. The bubbles advected (entrained) basaltic melt into the rhyolite in the form of filaments. The entrained melt in turn provided channels of low viscosity through which further bubbles followed. Consequently, repeated replenishment of the basaltic filament occurred, which stands in contrast to the conventional understanding of magma mixing filaments which were tacitly thought to never replenish (Wiesmaier et al. 2015).

In addition to bubbles, crystals and xenoliths moving relative to convective flow may also increase the efficiency of mingling/mixing of end-member silicate melts. Mafic enclaves hosted in felsic rocks can incorporate xenocrysts from the silicic host magma, demonstrating the potential complex interactions between melts and crystals during magma mixing (Wiesmaier et al. 2011). Feldspar megacrysts from the felsic host magma occur in disequilibrium in mafic enclaves and show their mobility across the felsic–mafic magma interface as observed in plutonic (Erdmannsdörfer 1946; Reid Jr. et al. 1983; Vernon 1986; Blundy and Sparks 1992; Weidendorfer et al. 2014) and volcanic rocks (Freundt and Schmincke 1992; Tepley et al. 1999; Troll and Schmincke 2002; Troll et al. 2004). Additionally, xenolith blocks from the roof of the magma chamber can sink through magma interfaces (Vernon 1986; Irvine et al. 1998). As the crystals and xenoliths move across the magma interface, they contribute actively and/or passively to the mingling/mixing process. A passive contribution is provided by a coupling of the crystals and xenoliths to the convective flow in a magma chamber, caused by thermal and compositional gradients. An active contribution can be driven by the object itself due to its density contrast, rising or sinking relative to the surrounding magma.

Particles settling through density-stratified liquids have been described in the fluid-dynamical and chemical engineering literature. As a sphere approaches a deformable interface, mathematical approaches have been employed to show that the viscosity contrast and the interfacial tension are the major influences on the deformation of the interface and subsequent entrainment of one liquid into the other (Lee and Leal 1982; Geller et al. 1986). High interfacial tensions of immiscible liquids inhibit the deformation of the interface and the movement of the sphere through it. With decreasing interfacial tension, the interface starts to be deformable, but the deformed interface area is very large, minimising the curvature of the interface. As the sphere finally moves through the interface, a tail behind the sphere rapidly thins out as the interface tries to minimise its energy. The film of liquid in front of the sphere drains away quickly, also due to interfacial tension forces (Geller et al. 1986). In the absence of interfacial tension, this thin film is more stable and the tail of liquid dragged behind the sphere stays constant and its radius decreases only marginally, resulting in significantly more efficient mingling (Geller et al. 1986).

In analogue experiments with two immiscible liquids at low Reynolds numbers and a viscosity ratio of 40 (upper to lower fluid), a Teflon particle approaching an interface has been shown to slow down significantly before passing through the interface (Manga and Stone 1995). Interfacial tension causes the liquid tail dragged behind the sphere to break up into small drops, reducing the interface energy (Manga and Stone 1995). If the liquids are miscible, the tail remains coherent (Srdic-Mitrovic et al. 1999) as, at high temperatures, interfacial forces are very transient and rapidly diluted by diffusional smearing (Mungall 1994; Lacaze et al. 2010). The falling sphere reaches the Stokes’ terminal velocity in the upper liquid and slows down close to the interface for prolonged residence time (Camassa et al. 2009, 2010). In summary, the mingling effect of a falling sphere is much more significant if the two liquids are miscible, where the interfacial tension is small and transient.

The aim of this study is to investigate the contribution of an object moving across the silicate melt interface in order to estimate its relevance in magma mingling/mixing processes independent of whether it moves actively or passively. We report the results of high-temperature particle settling experiments with platinum particles settling through stratified silicate melts of contrasting viscosity. At our experimental conditions (1450 °C, atmospheric pressure), the two melts have a viscosity contrast of three orders of magnitude, as in earlier magma mixing experiments using the same natural starting materials (Morgavi et al. 2013a, b, c). We have documented the mingling effects of the settling particle with X-ray microtomography of the quenched experiments. The chemical hybridisation was subsequently documented by the analysis of major elements across the deformed silicate melt interface. We compare the effectiveness of the chemical hybridisation of the rhyolite filament in a larger volume of basalt with recent experiments of a basalt filament entrained into a larger volume of rhyolite by bubbles (Wiesmaier et al. 2015).

## Methodology

### Glass preparation

The basalt and rhyolite used in the experiments are natural samples from the Bruneau–Jarbidge eruptive centre in the Snake River Plain (Idaho, USA). The rhyolite belongs to the unit V of the Cougar Point Tuff and the basalt to the Mary’s Creek basalt (Bonnichsen et al. 2008; Cathey and Nash 2009; Morgavi et al. 2013a). These materials were chosen for their very high viscosity contrast at high temperatures (4 × 10^3^) which should severely inhibit mixing. Unweathered rock samples were crushed and high-speed-milled. The rhyolite powder was melted at 1600 °C, and the basalt was melted at 1450 °C. The melts were stirred in a concentric cylinder apparatus (Dingwell 1986) for 60 and 5 h, respectively, to allow for complete degassing and to obtain bubble-free glasses after quenching. The basalt was prepared directly in the experimental crucible to avoid trapping of air between the glass and the crucible walls by transferring from one crucible to another. The surface of the basalt in the crucible was ground flat and polished on a lathe. After stirring the rhyolite, a spheroidal platinum particle with a 2.7 mm diameter was placed on the top surface of the melt, which was quenched as soon as the particle was observed to be covered by rhyolite. The particle-containing rhyolitic glass was then cored out of the synthesis crucible and machined into a cylinder, which was polished and placed on top of the basalt.

### Experimental set-up

**Fig. 1:**
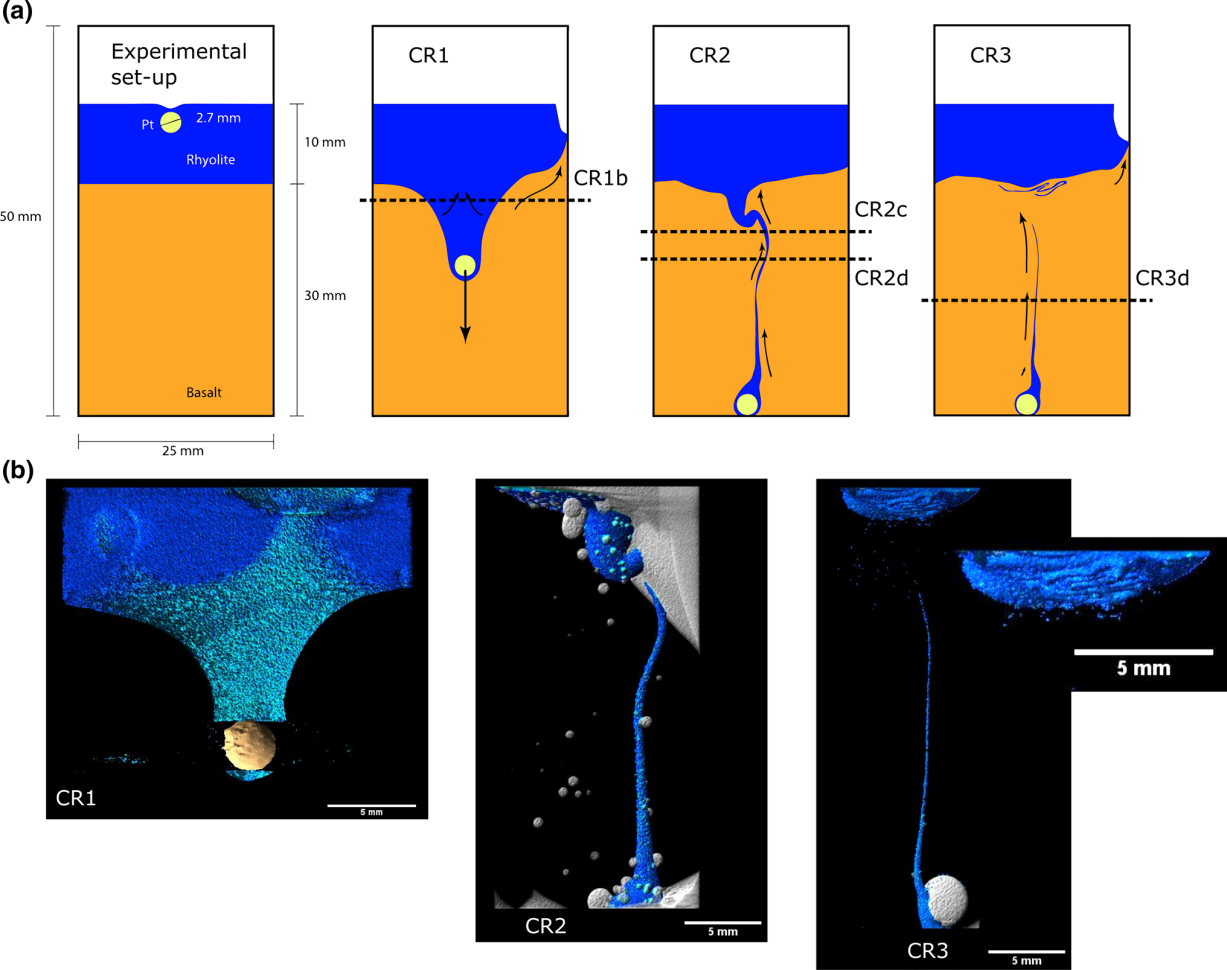
**a** Drawings show the experimental set-up and a schematic summary of the three experiments. Prior to the experiments, the platinum particle was placed 7 mm above the interface inside the rhyolite. Air could only be trapped at the basalt–rhyolite and rhyolite–platinum crucible interfaces. Drawings CR1, CR2 and CR3 show the quenched experiments of the time series at 45, 77 and 120 min, respectively. **b** The X-ray microCT images show the rhyolite in *blue*; the basalt is transparent in order to see internal structures. CR1 includes the Pt particle which causes artefacts around it, disturbing the visualisation of a continuous layer of rhyolite around the particle. Above the interface, outlines of bubbles in the rhyolite can be seen, dragging basalt with them. In CR2 and CR3, air is visualised in *white colours*. The basalt in CR2 was not entirely bubble free compared to CR3 which has no bubbles in the basalt. The only bubbles in the experiment are along the interface and the filament, including a large one at the base of the crucible

The experiments were performed in a cylindrical Pt crucible (Pt_80_-Rh_20_) with a height of 50 mm and an inner diameter of 25 mm. The crucible was filled with basalt to a height of approximately 30 mm plus 10 mm of rhyolite above that. Before the experiment, the rhyolite-embedded Pt particle was located ca. 7 mm above the interface between basalt and rhyolite (Fig. 1). Three experiments were run at 1450 °C and then quenched in air at room temperature. The experiments were run for 45, 77 and 120 min, respectively. The second and the third experiment were kept at 1000 °C for 10 min before quenching to avoid cracking of the basalt around the platinum particle as observed in the first experiment run for 45 min. At 1450 °C, the basalt had a viscosity of 3 Pa s and the rhyolite of 1.2 × 10^4^ Pa s. The calculated densities are 2.69 g/cm^3^ (basalt) and 2.29 g/cm^3^ (rhyolite) (Lange and Carmichael 1987). The Stokes’ terminal velocities ($v_{s} = \frac{{2\left( {\rho_{p} - \rho_{f} } \right)gr^{2} }}{9\mu }$, where *ρ*_*p*_ and *ρ*_*f*_ are the particle and melt densities, *μ* is the dynamic viscosity of the melt, *r* is the particle radius and *g* is the gravity constant, Happel and Bryne 1954) were calculated for an ideal platinum sphere in the basalt and the rhyolite neglecting the effects of mingling and the interface, as well as imperfections in the shape of the Pt spheroid. The calculated terminal velocities are 7 × 10^−6^ m/s in the rhyolite and 3 × 10^−2^ m/s in the basalt. The interface reduces the settling velocity of the particle in the basalt as the rhyolite tail exerts buoyancy and volume shear forces on the particle (Camassa et al. 2009, 2010). For these maximum settling velocities, the Reynolds numbers ($Re = \frac{{\rho v_{s} L}}{\mu }$, where *ρ* is the melt density, *v*_*s*_ is the velocity, *L* is the particle diameter and *μ* is the viscosity, Reynolds 1883) are 3.6 × 10^−12^ in the rhyolite and 7 × 10^−5^ in the basalt so that laminar flow can be assumed for all experiments.

### X-ray microCT

Run products were cylinders of experimental glass cored from the Pt crucibles. X-ray microcomputed tomography (microCT) images of the run products were obtained at the IMETUM institute, Garching, Germany using a General Electric v|tome|x s© device with a nanofocus X-ray tube. For each experiment, 1000 images were taken at 80 kV, 250 µA with 333-ms exposure time. Beam hardening was reduced with a 0p2VA filter for experiment CR1 resulting in a voxel size of 34.5 µm and a 0p6Cu filter for the experiments CR2 and CR3 with voxel sizes of 40 µm. The image sequence was reconstructed with VGStudio MAX© and subsequently rendered and analysed in the MATLAB-based script Tomoview.

### Major element analysis

Following microCT imaging, the experiments were cut into sections for chemical analysis. Major elements were analysed on a JEOL JSM6400 SEM with an Oxford Link ISIS Pentafet EDS system at the Centre for Advanced Microscopy at the Australian National University. The system was calibrated for quantitative major element analysis with 15 kV accelerating voltage, 1 nA beam current and 60 s counting time. Individual measurements were made at a magnification of 5000× over an area of 432 µm^2^ resulting in an effective beam current density of 2.3 × 10^−3^ nA/µm^2^ preventing Na-loss (Wykes 2014). The areas of raster measurements were aligned next to each other resulting in a spatial resolution ranging from 19 to 25 μm. The standards used were Si, sanidine; Ti, rutile; Al, albite; Fe, haematite; Mn, rhodonite; Mg, periclase; Ca, diopside; Na, albite; K, sanidine and P, apatite. The calibration was checked analysing a range of glass standards yielding an accuracy which is better than 3 %.

## Results

### X-ray microCT

**Fig. 2:**
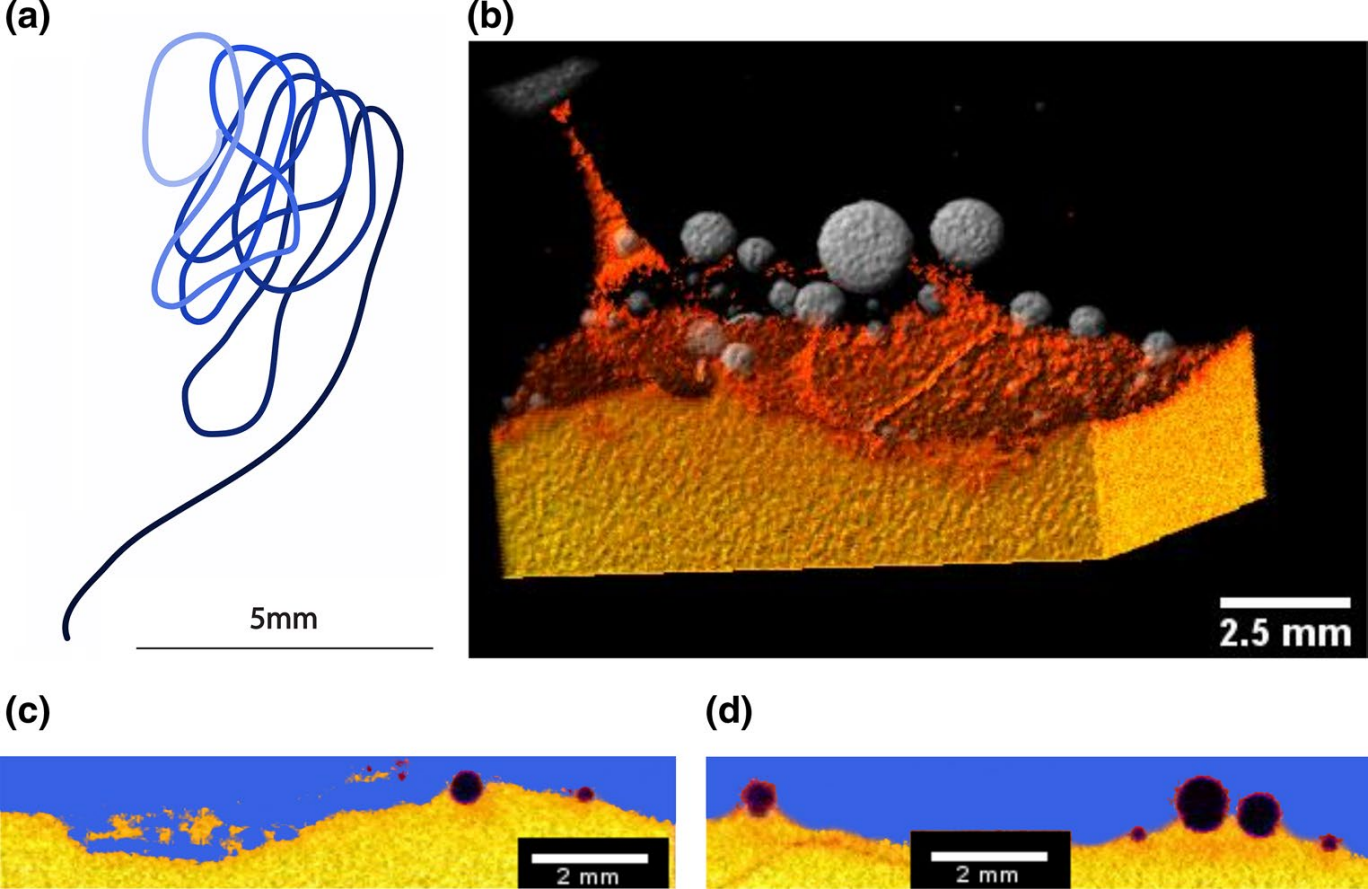
Observations are from experiment CR3. Rhyolite is shown in *blue* and basalt in *yellow* and *orange colours*. **a** Pile of coiled rhyolite filament below the interface with a total length of 70 mm. The shading from *light* to *dark* represents the position below the interface from *top* (*black*) to the *bottom* (*light blue*). **b** Bubbles originating at the interface start rising into the overlying rhyolite and drag basalt filaments with them (*yellow*/*orange*). **c** Cross section through the filament pile. **d** Bubbles moving across the interface do not have ideal spherical shapes. The bubble radii and surface curvatures change as they move across the interface

The colours of the tomography images (Figs. 1, 2) correspond to the X-ray attenuation of the different glasses in the experiment and are a qualitative measure of chemical hybridisation. Differences in colour shades are due to small gradients in the X-ray attenuation towards the edge of the segmented volumes. The time series of three experiments allows the observation of the mingling process with time (Fig. 1). Absolute volume measurements of the rhyolite dragged into the basalt were not possible because the platinum particle caused unresolvable artefacts in the CT image of experiment CR1. Rhyolite surrounding the platinum particle is not visible in the 3D reconstruction, as the high X-ray attenuation of the Pt sphere saturated the greyscale values with a concentric artefact. This artefact is expressed as a gap in the rhyolite film around the Pt sphere in Fig. 1b. Furthermore, the entire volumes of rhyolite were not retrieved from the base of the crucibles of experiments CR2 and CR3 due to fracturing during quenching.

## Experiment CR1 (45 min)

The experiment was quenched when the platinum sphere had sunk about one centimetre into the basalt, dragging rhyolite liquid with it. The platinum sphere is completely encased in a film of rhyolite with a measured thickness of 0.75 mm. The rhyolite film does not seem to drain away from the sphere due to its high viscosity, and the liquid tail following it shows no necking. Draining of the rhyolite away from the sphere would allow it to break through the interface between rhyolite and basalt without dragging any more rhyolite melt into the basalt. During the settling process, the Pt particle is always only in contact with rhyolite melt and never with basalt melt or air bubbles from the interface. Consequently, the wetting properties of the Pt particle remained constant throughout the experiments.

## Experiment CR2 (77 min)

The Pt sphere reached the bottom of the crucible and was not recovered with the experimental charge drilled from the crucible (Fig. 1). The rhyolite on top is connected by a filament with the Pt sphere at the base of the crucible. The sphere remained attached to the rhyolite filament during the experiment. At the thinnest point, the filament has a diameter of 0.5 mm. The filament is observed to bend back up to the initial interface, probably a buoyancy effect from the lower density of the rhyolite. This process may have been accelerated by several bubbles attached to the rhyolite filament.

## Experiment CR3 (120 min)

The buoyancy of the rhyolite filament is better resolved in the longest experiment of 120 min run-time. Here, a finely coiled filament has piled up below the basalt–rhyolite interface, forming figures of eight. The rhyolite filament in the basalt broke up in the middle, probably because the thinned down diameter from stretching of the filament became too small (<0.2 mm). The total length of the filament, including the pile, has been measured to a total length of 90 mm (Fig. 2), which is a threefold increase compared to the vertical distance from the base of the crucible to the interface. The length of the filament in the pile alone is 70 mm. The volume of the rhyolite filament in the pile is ~5 mm^3^, and the volume of the entire pile is ~10 mm^3^. The pile consists of comparable volumes of intermingled rhyolite and basalt which were not fully hybridised. Based on the greyscale values of the tomography, the chemical gradient at the undisturbed interface has a vertical extension of 0.3–0.4 mm between basalt and rhyolite. The mingled filament pile has a thickness of 1.6 mm. This is a significantly thicker hybrid region compared to the undisturbed interface where chemical diffusion was the only hybridisation mechanism.

## Bubbles at the interface

The basalt cylinders were molten and homogenised in the experimental crucibles. Hence, no air was trapped between the crucible and the basalt and the basalt melt was as bubble-free as possible (Fig. 1). In the run product of experiment CR2, several bubbles were observed in the basalt (diameter <1 mm), probably from incomplete degassing during pre-experimental remelting. According to Stokes’ law, these bubbles moved a maximum of 2 mm at the experimental conditions. In contrast, experimental glasses from CR1 and CR3 showed bubbles that have formed in situ at the basalt/rhyolite interface, i.e. syn-experimental.

The largest bubbles have diameters of 2 mm and sit on the interface of basalt and rhyolite around the platinum sphere, with a thin film of rhyolite remaining between bubbles and platinum sphere. Smaller bubbles ranging from 0.5 to <0.1 mm occur at the initial rhyolite/basalt interface but rarely seem to have moved far into the overlying rhyolite. All of these small bubbles seem to have been formed at the interface between the basalt and the rhyolite cylinder during the set-up of the experiment as trapped air. The exception are three large bubbles (>2 mm in diameter) at the rim of the experiment which have dragged significant volumes of basalt into the rhyolite (Wiesmaier et al. 2015) and seem to have originated at the interface between the rhyolite glass cylinder and the surrounding platinum crucible. The tomography of experiment CR3 shows very few bubbles along the filament, suggesting that the main buoyant driving force was the rhyolite itself and not bubbles dragging it upwards.

At the initial rhyolite/basalt interface, larger bubbles (>2 mm) are observed to have risen into the rhyolite, starting from the interface. Smaller bubbles (diameter <0.5 mm) appear to have entered the filament formed by a larger bubble (Fig. 2). The tomography shows a gradient of X-ray attenuation between the basalt filament and host rhyolite, indicating significant syn-experimental diffusional equilibration between basalt filament and surrounding rhyolite. This stands in contrast to the rhyolite filament (formed in the basalt by the falling Pt sphere), which lacks diffusional gradients of similar magnitude in the tomography. As the bubbles migrate through the interface, they change shape due to changes in surface tension between the bubble/basalt and the bubble/rhyolite interfaces (Fig. 2). The parts in contact with rhyolite have a smaller radius than the parts in contact with basalt. At the level of the interface, the surface curvatures of the bubbles change accordingly. The surface tension of dry rhyolite to air is higher than that of dry basalt to air (Bagdassarov et al. 2000). Upon passing of the bubble into the rhyolite, no basalt film is preserved around the bubble, in contrast to the settling platinum particle, which remained fully encased in rhyolite within the basalt. Only a small section of the bubble stays in contact with the basalt filament below and acts as the connection between the bubble and the filament. Bubbles were observed to always remain attached to their basalt tails within the experimental timescales.

### Major elements

**Fig. 3:**
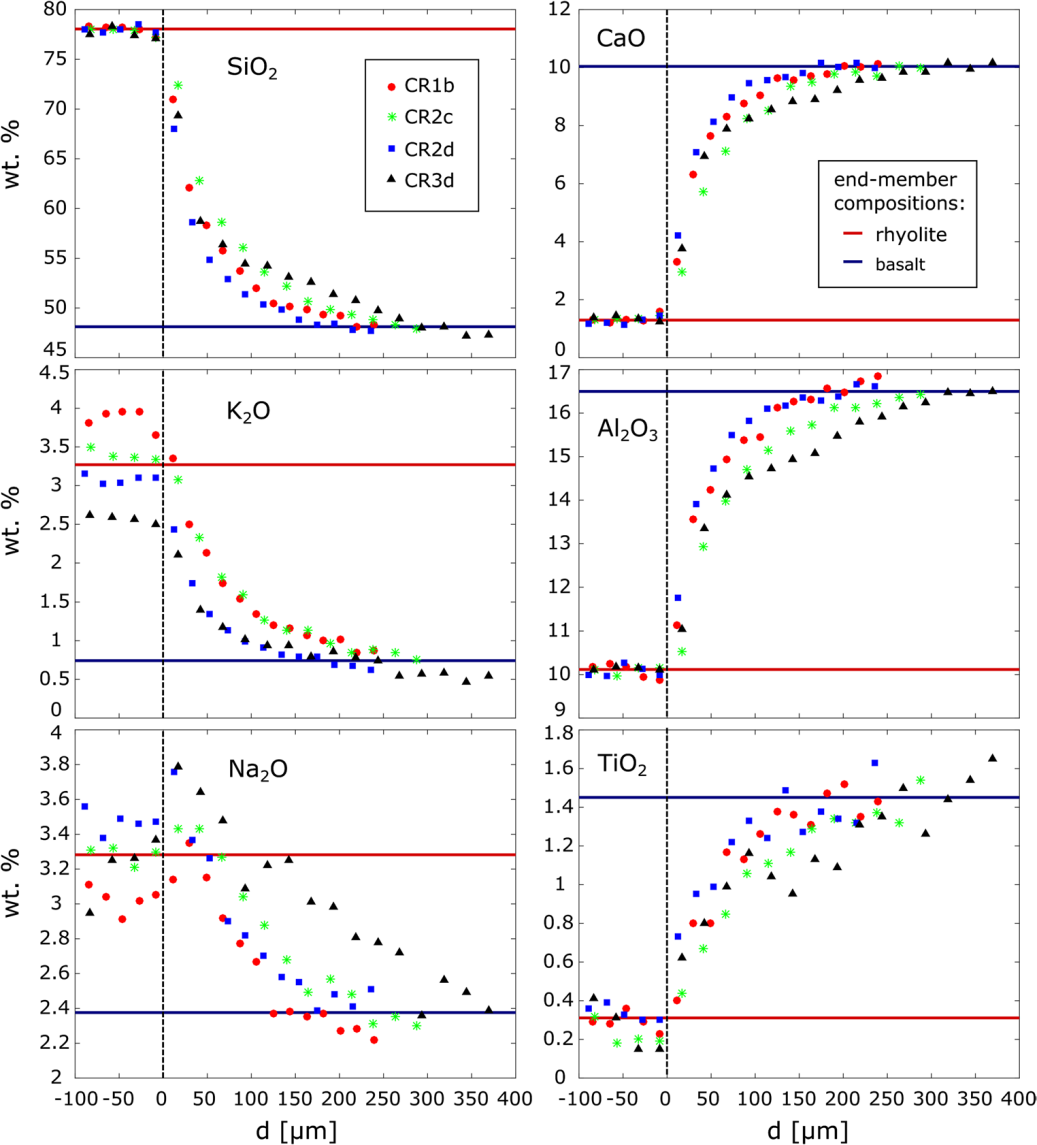
Chemical profiles of major elements measured in the experiments. The *red bar* shows the end-member composition of the rhyolite and the *blue bar* the end-member composition of the basalt, as measured in the experimental samples. The profile CR3d shows a step at 200 μm distance from the interface in the basalt. This is due to buoyant backflow of the rhyolite filament replenishing the surrounding basalt. Sodium and Ti show uphill diffusion. Variations in the K_2_O and Na_2_O concentrations in the rhyolite end-member are due to different durations of melt homogenisation at 1600 °C, resulting in varying degrees of volatile loss, and analytical uncertainties

On all run products, major element transects were measured at the interfaces of the rhyolite filament and surrounding basalt. To avoid an excessive amount of data points and machine time, the interior of filaments was not analysed due to their internal chemical homogeneity demonstrated by their constant X-ray attenuation. The diameters of the filaments range from 350 µm in experiment CR3 to 3 mm in experiment CR1. The resolution of the measured profiles is approximately 20 µm. The profiles are laterally asymmetric for all chemical elements with much longer chemical gradients in the basalt than in the rhyolite (Fig. 3). Although the position of the original interface is unknown, the asymmetric shape of the profiles suggests that it is near the change of profile slopes as shown in Fig. 3. The extent of chemical hybridisation has not progressed far at the given short experimental timescale showing the onset of chemical mixing. In all three experiments, the rhyolite filament retained its end-member composition, only a minor concentration gradient in the rhyolite develops with increasing time in the experiments CR2 and CR3 compared to the basalt. Despite different experimental run-times, the concentration profiles of the three experiments have comparable lengths and shapes. Potassium and Na show significant variations in their absolute concentrations in rhyolite in the different profiles and experiments analysed.

We have chosen four major element profiles to exemplify the chemical hybridisation of the three experiments. The positions of the sections in which the profiles were measured are shown in Fig. 1a. Silica is used to describe the shape of the diffusional profiles as it is the slowest diffusing element (Dingwell 1990) and has a large chemical gradient between the two end-members of 30 wt%. In profile CR1b, the gradient between the two SiO_2_ end-member compositions is approximately 200 µm. It is exemplary of other profiles measured in experiment CR1 with less spatial resolution. CR3d on the other hand, shows the longest diffusional profile as expected, as it was the longest lasting experiment. Interestingly, the shape of the profile is not ideal, showing a hump in its tail in the basalt. The profile flattens at 100 µm distance from the rhyolite end-member and steepens again at 250 µm before reaching the basalt end-member composition. The two profiles of experiment CR2 (CR2c and CR2d) were measured in different sections across the filament (Fig. 1). Their shapes are surprisingly different. At a distance of 100 µm from the rhyolite end-member, their compositions vary at about 5 wt% SiO_2_. Sodium shows a significant increase and Ti a decrease in their concentrations at the position where SiO_2_ reaches its rhyolite end-member concentration. These deviations exceed the end-member concentrations, and the Na_2_O concentration reaches a maximum where the SiO_2_ concentration is ~70 wt%. Both elements experience uphill diffusion. This suggests that their diffusional behaviour is coupled (Mungall et al. 1998).

In experiment CR3, the Na uphill diffusion is most pronounced. The Na_2_O concentration decreases into the rhyolite filament which has rhyolite end-member concentrations of all other major elements. A section across the entire rhyolite filament would show an M-shaped Na_2_O profile reaching a minimum within the filament. This minimum is significantly lower than the initial Na concentration. Conversely, the Ti profile has a maximum within the filament.

**Fig. 4:**
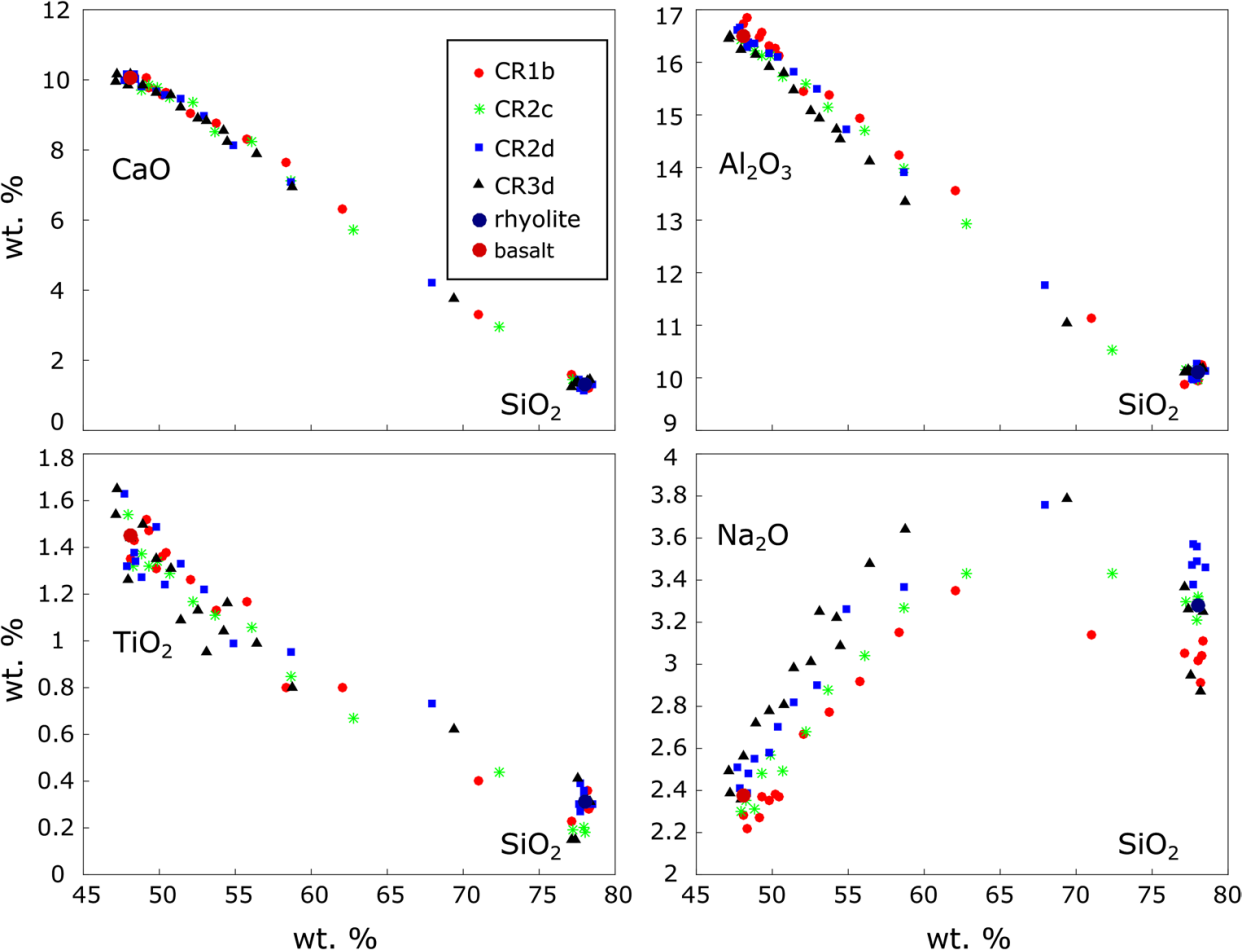
Inter-elemental plots of CaO, Al_2_O_3_, TiO_2_ and Na_2_O versus SiO_2_. The nonlinearity of the TiO_2_ and Na_2_O profiles is due to uphill diffusion and chemical fractionation during diffusion. Both elements diffuse significantly faster than Si and all other elements and reach a hybrid composition sooner

According to ideal hybridisation, inter-elemental plots of mixing melts should show linear correlations between different chemical elements [see Eq. (1)]. Nonlinear relationships are evidence for diffusive fractionation of different chemical species (De Campos et al. 2008). Some chemical elements such as Na can diffuse significantly faster than others and reach a hybrid concentration sooner (Fig. 4). They decouple from the diffusion of other elements which are slower. Inter-elemental plots of fast versus slow elements show S-shaped patterns. Inter-elemental plots of chemical elements which diffuse at similar rates in our experiments show linear correlations, but they are not symmetrical, as the compositional space near the basalt end-member is much denser populated than the space near the rhyolite end-member.

## Discussion

### Viscosity contrast in the experiment and in natural systems

We conducted our experiments at 1450 °C which is much higher than temperatures in natural systems. In a felsic magma reservoir, the typical temperatures of the silicic magma are expected to be around 800 °C, while replenishing basalt is expected to have temperatures of 1100–1200 °C (Jellinek and Kerr 1999; Troll and Schmincke 2002). At this temperature difference, the viscosity contrast between our experimental rhyolite and basalt melts is eight log *η* (Pa s) units (Giordano et al. 2008). As the magmas thermally equilibrate, the viscosity contrast reduces to a factor of 4000. The viscosity contrast between the two melts in thermal equilibrium is roughly constant between 800 and 1450 °C, so that the viscosity contrast expected in a melt dominated natural magma chamber with no significant thermal contrast approaches that of our experimental set-up at far higher temperatures. The high experimental temperatures have the advantage of making observations at reasonable experimental time scales and at extreme viscosity contrasts. Additionally, the high experimental temperatures compensate for the lack of water in the experimental melts. In order to reach the experimental viscosities at a lower temperature of 1000 °C, the end-member rhyolite and basalt would require more than 4 wt% of H_2_O.

The process described in this work requires that the two interacting magmas are comparably free of particle interlocking in order to permit efficient particle motion. Such a couple of sub-liquidus magmas form most likely in nature when heat from fresh basaltic magma remobilises stagnant felsic melt (cf. Wiesmaier et al. 2011). Remobilisation of felsic magma in nature depends on the heat budget of both involved magmas and their respective extrinsic parameters. This has been confirmed in many case studies where mafic underplating and remobilisation of felsic melt have been found to trigger large felsic eruptions (Turner 1980; Campbell and Turner 1986; Turner and Campbell 1986; Eichelberger and Izbekov 2000; Izbekov et al. 2004; Wark et al. 2007) and in theoretical approaches concerning the reactivation of crystal mushes (Girard and Stix 2009; Huber et al. 2011).

Due to this thermal bracket of favourable temperature, and therefore viscosity conditions, the process of particle settling must accelerate with progressive thermal equilibration of the two magmas. Termination of particle settling will occur either instantly by eruption or gradually by heat loss and simultaneous crystallisation of the involved magma. Elapsed time from thermal equilibration of two magmas to eruption has been estimated as months–years (Druitt et al. 2012; Reid 2014). Gradual heat loss of igneous systems may span a wide range of timescales, up to hundreds of thousands of years (Best 2002).

The intrinsic timescale of particle settling in nature is governed by density contrast between particle and magma. Whereas the high density of the platinum particle allowed for short experimental durations, the density contrast in natural systems will be reduced, implying a decreased terminal settling velocity. However, this is offset by the comparatively large time window in natural settings. Particle settling under Stokes’ law is furthermore influenced by particle size, so that crystal size distributions that comprise large crystals favour particle settling induced mixing. Additionally, as thermal expansivity of rhyolite melt is larger than that of particles within the melt, the density contrast increases with T and thus further facilitates particle settling (e.g. Bagdassarov and Dingwell 1994).

### Interpretation of 3D images

Our experimental goal was to observe the behaviour of the rhyolite filament after it was dragged into the basalt. We estimated the experimental timescales based on Stokes’ terminal settling velocities of the platinum particles in the melts. They are 7 × 10^−6^ m/s and 3 × 10^−2^ m/s for the rhyolite and basalt melts at 1450 °C, respectively. The particle had to pass through 7 mm of rhyolite and 30 mm of basalt before reaching the bottom of the crucible. Assuming terminal settling velocities, the particle would have passed through the rhyolite layer in 1000 s and through the basalt in 1 s. To allow for acceleration of the particle and passage through the interface, the timescale of the first experiment (CR1) was set to 45 min. The timescales of the other experiments were selected after the X-ray tomography of experiment CR1 was evaluated and we observed that the particle had not yet reached the bottom of the crucible.

X-ray microCT imaging reveals efficient mingling of the rhyolite and basalt melts. The platinum particle dragged more than three times its own volume of rhyolite into the basalt. The liquid tail behind the sphere shows no necking in experiment CR1 due to the transience of interfacial tensions (Geller et al. 1986). The high viscosity of the rhyolite melt probably slowed drainage of the thin film around the sphere, so that the film is preserved on the comparatively short experimental run-time despite the rhyolite’s buoyancy. Significant buoyant rise of rhyolite apparently only began once the sphere reached the base of the crucible, which stopped the downward drag force (observed in experiments CR2 and CR3). According to Stokes’ law, the terminal settling velocity of the platinum sphere in basaltic melt is five orders of magnitudes larger than the upward terminal settling velocity of a spherical drop of rhyolite of the same size. Buoyant rise of rhyolite is thus limited as long as the platinum particle is moving, and the rhyolite is continuously stretched in that process as observed in the microCT images.

Once the rhyolite flows back up to the interface, the filament behaves like a liquid rope. The competence of the filament and the lack of disintegration into the surrounding basalt suggest transient interfacial tension effects between the rhyolite and the basalt (Mungall 1994; Lacaze et al. 2010). The filament behaves like a liquid rope and coils up below the interface forming “figures of eight”. Liquid rope coiling is in essence a buckling instability caused by longitudinal compressive stress along the filament as it approaches the interface (Barnes and Woodcock 1958; Barnes and Mackenzie 1959; Ribe 2004; Maleki et al. 2004; Ribe et al. 2006a, b, 2012; Habibi et al. 2006, 2010). Coiling frequency varies with the properties of the liquid rope, which creates different coiling regimes as a function of viscous, gravitational and inertial forces (Ribe et al. 2012). The coiling frequency generally increases with increasing fall height of a liquid rope, which consists of a tail deformed by stretching and a coil deformed by bending (Habibi et al. 2010). At small heights in the viscous regime, the tail is very short and viscous forces counteract bending of the liquid rope (filament). With increasing fall heights, the tail makes up a bigger part of the length of the liquid rope (Ribe et al. 2012). The viscous regime is superseded by gravitational and inertial forces with increasing fall heights and the tail will consequently be dominated by gravitational stretching. At intermediate heights in the inertia-gravitational regime, several coiling frequencies can occur for the same fall height. In this multivalued range, the coiling changes its sense of rotation multiple times and in addition to the rotating coil, the tail behaves like a conical pendulum (Ribe et al. 2012). During the change of the rotational sense, the coiling forms figure of eight patterns as observed in experiment CR3 (Ribe et al. 2006a, b, 2012; Habibi et al. 2006). In the inertia-gravitational regime, the properties of the rhyolite filament are therefore dominated by strong buoyant stretching of the tail, and interference between the coiling at the interface and the conical pendulum like motion of the tail.

### Chemical hybridisation

Buoyant backflow of rhyolite initially dragged down by the platinum particle is not just recorded by the X-ray tomography but also by the length and shape of the chemical profile in the basalt. A step observed in the chemical profile of experiment CR3 at 200 μm distance from the interface is interpreted as the result of a second pulse of rhyolite moving through the basalt. The initial pulse was caused by the rhyolite dragged downwards by the platinum particle. Once the particle stopped at the base of the crucible, the rhyolite started to rise buoyantly from the base of the crucible back through the same volume of basalt, causing a second pulse of rhyolite moving through the basalt.

The filament which is coiled up back at the initial interface in experiment CR3 essentially builds a new layer in between the basalt and the rhyolite. That layer is significantly wider than the diffusional interface measured in run products CR1 and CR2 (1.6 vs 0.3 mm). The following simple calculation shows that the particle-induced mingling has significantly increased the chemical hybridisation at the interface. The composition of the completely hybridised filament pile can be calculated based on a simple two end-member mixing equation (De Campos et al. 2008):

1 $$C_{\text{hybrid}} = C_{\text{rhyolite}} x + C_{\text{basalt}} \left( {1 - x} \right)$$

where *C*_hybrid_ is the calculated concentration of a given element in the hybrid, *C*_rhyolite_ and *C*_basalt_ are the measured end-member concentrations and *x* is the volumetric proportion of the rhyolite end-member in the pile, which is 1:1 according to the tomography estimates in the filament pile. A completely hybridised filament pile would have an andesitic composition and form a significantly wider hybridised zone at the interface between the basalt and overlying rhyolite compered to diffusion alone. A tomography slice across this transient melange of basalt and rhyolite (Fig. 2c) shows similarities with chaotic mixing as it is made of stretched and folded rhyolite filament in basalt.

In the experimental run products CR2 and CR3, we observe the backflow of the rhyolite filament from a rhyolite reservoir, surrounding the platinum particle at the base of the crucible and stretching upwards up to the initial interface. The filament moves along the same axis that it descended previously, by stretching the rhyolite reservoir surrounding the particle. Assuming that the reservoir is made of a 0.75-mm-thick layer of rhyolite (as observed in experiment CR1, Fig. 1) around the platinum particle with a diameter of 2.7 mm, the volume is 28.5 mm^3^ and the interface area between this rhyolite volume and the surrounding basalt is 55 mm^2^. A cylindrical filament of the same volume, a length of 30 mm and a diameter of 0.55 mm would have an interface with the basalt of 106 mm^2^. Therefore, the volume at the base of the crucible has a surface area half of that of the filament and the same volume and thus did hybridise less, and the material flowing back upwards has a composition close to the rhyolite end-member. The backflow causes a continuous replenishment of fresh rhyolite in the central part of the basalt volume. Such a backflow is an additional component to the mingling mechanisms of stretching and folding. Another way of filament replenishment was observed by Wiesmaier et al. (2015), where a basalt filament was continuously replenished by bubbles moving through it and dragging more fresh basalt into it.

### Comparison with bubble-induced mixing

Recently, Wiesmaier et al. (2015) demonstrated that gas bubbles may advect portions of low-viscosity melt into an overlying high-viscosity melt even at high viscosity contrasts. Their experiments were performed at the same conditions and with the same material as the ones that we present here. The comparison of particle-induced mixing with bubble-induced mixing is thus pertinent. The main differences are directional and rheological. In the bubble case, low-viscosity filaments are dragged upwards into an overlying high-viscosity liquid, whereas in the dense particle case, high-viscosity filaments are advected downwards into an underlying low-viscosity liquid. Viscosity contrast is a significant rheological factor in the mingling behaviour (cf. Manga and Stone 1995). Although the total viscosity contrast is equal, the inverted viscosities (the filament is of high instead of low viscosity) results in considerably different filament behaviour.

Considering the effect of bubble mixing, our experiments were much shorter than the experiment presented by Wiesmaier et al. (2015) which ran for 180 min. In their experiment, bubbles originating at the interface and bubbles from the basalt had enough time to penetrate the basalt/rhyolite interface and drag basalt into the overlying rhyolite. Our experiments allow an extrapolation of their observations to shorter experimental timescales. Wiesmaier et al. (2015) have observed the coalescence of basalt filaments in the rhyolite and the formation of a low-viscosity “tunnel” through which multiple bubbles can rise. In our experiments, this coalescence has not occurred yet. Individual bubbles start dragging small amounts of basalt into the rhyolite evidenced by many small filaments (Fig. 2). The shape of the filament is considerably different in comparison with the rhyolite filaments formed by the Pt particle. The bubbles can move through the interface, at least partially, before they start dragging basalt with them. As they move through the interface, they change their shape due to changing interfacial tensions with the two different melts. Only a small section of the bubble continues to stay in contact with the basalt and forms the connection with the filament. This is different compared with the rhyolite filament which continues to completely enclose the particle while sinking into the basalt. This is due to the different viscosities which prevent the rhyolite from flowing away from the particle on the timescale of settling.

The coiling behaviour of the rhyolite filament within basalt was not observed with the basalt filaments within rhyolite. The coiling of the rhyolite filament was the consequence of buoyant backflow of the rhyolite after the downward dragging force of the platinum particle has ceased. Accordingly, the basalt should sink downwards after the bubbles cease to drag the filament upwards. This does not occur because additional bubbles follow through the filament and prohibit the sinking of basalt back to the initial interface. Additionally, the basalt filament is confined in surrounding high-viscosity liquid, whereas the rhyolite filament in the low-viscosity basalt was able to move without being constrained by the surrounding melt.

## Conclusions

Particle-induced magma mixing was demonstrated to occur between rhyolite and basalt melts at high temperatures and viscosity contrast. The mingling was induced by the negatively buoyant motion of a Pt particle, which settled from rhyolite into underlying basalt. The experiments demonstrated particle-induced mixing to function to viscosity contrasts of up to 4000. The X-ray tomographies of our time series of experiments allowed the visualisation of the magma interface shape evolution in time. The results show a high deformability of the interface and imply the absence of interfacial tension effects. Rhyolite which was initially mingled into the basalt flowed back to the initial interface, where it formed a considerable layer of hybrid material. This hybrid horizon due to mingling is four to five times thicker than a hybrid horizon formed by chemical diffusion alone might have been.

The movement of settling crystals and xenoliths can cause an increase of local mingling at the interface of mafic and felsic magmas. The buoyancy of the felsic magma works against the pull of the settling xenoliths and crystals. A limiting factor of the process is the density difference between the settling object and the dense underlying basalt. It needs to be greater than the buoyant upward force of the felsic magma. As the felsic filament moves back to the interface, it starts hybridising resulting in an intermediary composition which ultimately ends up at the initial interface. The whole process results in a wider hybrid layer between the end-members and increases the mixing efficiency of the system over all. We urge the further modelling and experimentation on these complex processes as well as the development of field tests of their relevance for mingled/mixed systems in nature.

## Electronic supplementary material

Below is the link to the electronic supplementary material.

Supplementary material 1 (AVI 6565 kb)

Supplementary material 2 (AVI 3343 kb)

Supplementary material 3 (AVI 1784 kb)

Supplementary material 4 (AVI 1898 kb)
